# Constructing ultra-stable, high-energy, and flexible aqueous zinc-ion batteries using environment-friendly organic cathodes†

**DOI:** 10.1039/d4sc00491d

**Published:** 2024-02-27

**Authors:** Chaojian Ding, Yonghui Wang, Chaobo Li, Jiawen Wang, Qichun Zhang, Weiwei Huang

**Affiliations:** a Hebei Key Laboratory of Applied Chemistry, Yanshan University Qinhuangdao 066004 Hebei China huangweiwei@ysu.edu.cn; b Department of Materials Science and Engineering, City University of Hong Kong Hong Kong SAR 999077 China qiczhang@cityu.edu.hk; c Center of Super-Diamond and Advanced Films (COSDAF), Department of Chemistry, City University of Hong Kong Hong Kong SAR 999077 China qiczhang@cityu.edu.hk

## Abstract

Due to their sustainability, environmental friendliness, high specific capacity, and rapid reaction kinetics, quinone cathodes have broad application prospects in aqueous zinc-ion batteries (AZIBs). However, conventional small-molecule quinone cathodes usually suffer from unavoidable dissolution, resulting in terrible cycling stability. Herein, based on a strategy of molecular structure optimization, calix[8]quinone (C8Q) is for the first time used as a cathode in AZIBs. By extending the structure of the classical small-molecule quinone cathode calix[4]quinone (C4Q), C8Q further adds four *p*-benzoquinone structural units, which significantly suppresses the dissolution of its discharge products and greatly improves the cycle stability of the cathode. Specifically, the C8Q cathode displays a discharge specific capacity of 207.2 mA h g^−1^ at 1 A g^−1^ and a long-life cycle stability (93 mA h g^−1^/10 A g^−1^/10000^th^). Even with a high active material loading of 11 mg cm^−2^, the Zn‖C8Q battery also exhibits high redox reversibility and remarkable electrochemical stability. Furthermore, the belt-shaped Zn‖C8Q battery has high stability and outstanding flexibility, indicating its promising application in flexible wearable electronic devices.

## Abstract 2

Aqueous zinc batteries using environment-friendly and sustainable quinone cathodes realize a long life cycle, high active mass loading, and excellent flexibility, showing its potential for application in wearable electronics.

## Graphical abstract

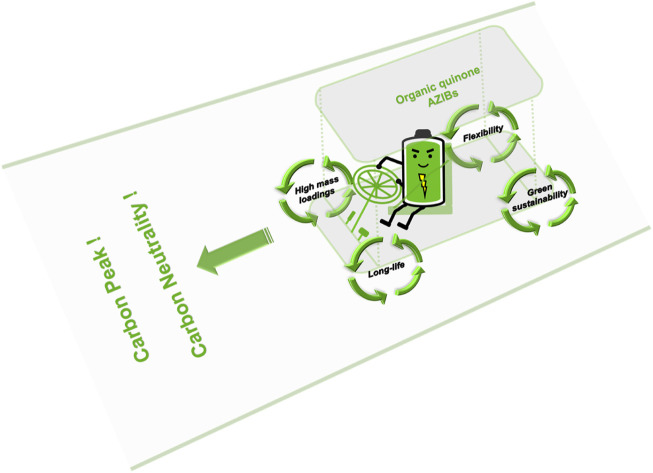

## Introduction

For a long time, developing novel cathode materials has been one of the research priorities for aqueous zinc-ion batteries (AZIBs).^1–3^ Initially, the exploration of electrode materials for AZIBs revolved around some conventional inorganic materials.^4–10^ However, the crystal lattice structure of inorganic materials tends to undergo irreversible distortion, collapse, and dissolution in Zn^2+^ storage and release, ultimately resulting in poor cycle stability of AZIBs.^11,12^ Unlike inorganic cathodes, the flexible structure of organic molecules endows organic cathodes with enhanced durability during repeated insertion/extraction of Zn^2+^. More importantly, organic cathodes are environmentally friendly, renewable, abundant in raw materials, and have high structural tunability, which makes them a research hotspot at present.^13,14^

Among various organic cathodes, quinone cathodes usually have wonderful electrochemical performances on account of their good electrochemical reversibility in the charge and discharge process.^15–18^ However, quinone cathodes (AQ, NQ, BQ, *etc.*) are deeply hampered by their solubility, causing their poor actual electrochemical performance.^19,20^ In 2018, C4Q was for the first time reported as a cathode for AZIBs. The strategy of cyclic polymerization of four benzoquinone structural units inhibited the dissolution of C4Q in aqueous electrolyte systems to a certain extent: the Zn‖C4Q battery delivered an initial capacity of 335 mA h g^−1^.^21^ However, during the subsequent cycle, the discharge products of C4Q (Zn_*x*_C4Q, *x* = 1∼3) exhibited a severe dissolution and shuttling phenomenon in aqueous electrolytes, leading the capacity of batteries to decay very fast. Although the dissolution and shuttling of active cathode materials were significantly improved after adopting the cation exchange membrane as the separator, this obviously increased the assembly cost of the Zn‖C4Q battery and weakened the competitiveness of the C4Q cathode in practical AZIBs. Nonetheless, this result has inspired us to further explore the application of quinone cathodes in AZIBs.

Up to now, many other methods have also been reported to solve the problem of the poor cycle stability of the above quinone cathodes, such as the combination of quinone compounds with mesoporous materials and activated carbon,^22,23^ modified separators,^24–26^ modified electrolytes,^27,28^*etc.* However, the external environment of active electrode materials is only considered, and the dissolution problem has not been fundamentally solved. Taking the molecule itself as the starting point, adopting a rational molecular structure design is undoubtedly a fundamental strategy for the dissolution problem of the small-molecule quinone cathode mentioned above.^29–32^

Herein, adopting a strategy of increasing the molecular weight to inhibit the dissolution of small-molecule quinone cathodes, we investigated the electrochemical properties of the C8Q cathode in AZIBs for the first time. With benzoquinone as the active structural unit, C8Q possessed a theoretical capacity of 447 mA h g^−1^. The further expanded long non-polar ring chain effectively inhibited the dissolution and shuttling of C8Q and its discharge products (Zn_*x*_C8Q, *x* = 1–4) in aqueous electrolytes, significantly improving the cycling durability of the electrode. Benefiting from these aforementioned characteristics, the C8Q cathode achieved a stable long–life cycle without using expensive Nafion membranes, whose cost is more than 17 times that of glass fiber membranes (Fig. S1†). In 3.0 M Zn(OTf)_2_, the C8Q cathode displayed an initial discharge capacity of 207.2 mA h g^−1^ at 1 A g^−1^ and an ultra-long cycle life over 10 000 cycles at 10 A g^−1^ (0.0059% capacity decay/cycle). In addition, structural optimization gives the C8Q cathode the advantages of high-loading capability and flexibility as well. The high-mass loading C8Q cathode (11 mg cm^−2^) and the flexible belt-shaped Zn‖C8Q batteries both showed excellent cycle stability, demonstrating their potential application in diversified energy storage areas.

## Experimental section

The source of all chemical reagents used can be found in the ESI.† The details about the synthesis, characterization, and electrochemical measurements of C8Q have been provided in the ESI (Scheme S1 and Fig. S2, S3, S4, and S18c†).^33,34^

## Results and discussion

### Solubility comparison between C4Q and C8Q

To understand the effect after the molecular structure optimization, a series of dissolution experiments for C4Q and C8Q were performed. In a two-week static dissolution experiment, the colour of 3.0 M Zn(OTf)_2_ electrolyte soaked with the C4Q cathode (pristine) gradually deepened (Fig. S6a†). Conversely, the colour of the electrolyte soaked with the C8Q cathode (pristine) barely changed over the two-week period (Fig. S7a†). Further analysis of the electrolyte was conducted by UV absorbance spectroscopy. In comparison to the blank sample, there was hardly any enhancement in the intensity of the absorption peak around 250 nm in the electrolyte soaked with the C8Q cathode (pristine), confirming the almost insoluble characteristics of C8Q in aqueous electrolytes (Fig. S6b and S7b†).

In addition to focusing on the solubility of the active molecules themselves, the tolerance of the discharge products of C8Q and C4Q in the aqueous electrolyte was also compared. In 3.0 M Zn(OTf)_2_ electrolyte soaked with the C4Q cathode (discharged to 0.20 V), the colour of the electrolyte was significantly deepened over time and the intensity of the corresponding UV absorption peak (250 nm) rose sharply. This unequivocally verified the high solubility of the discharge product of C4Q (Zn_*x*_C4Q, *x* = 1–3) in the aqueous electrolyte (Fig. S6c†). Remarkably, the considerable solubility of Zn_*x*_C4Q led to a severe shuttling effect in the actual charge–discharge process, thereby influencing the uniform deposition of zinc (Fig. S5†). Different from C4Q, the discharge products of C8Q (Zn_*x*_C8Q) still revealed ultralow solubility and good structural stability in aqueous electrolytes (Fig. S7c†).

In virtue of the high hydrophobicity of Zn_*x*_C8Q, the assembled Zn‖C8Q battery exhibited high stability as well in the visualized *in situ* dynamic charge–discharge dissolution experiments (Fig. S8†). In contrast, the dissolution and diffusion of C4Q and its discharge products (Zn_*x*_C4Q) were markedly more apparent in the actual process of the charge and discharge cycle under the influence of the internal electric field. This result suggests that reasonable molecular structure optimization can realize the low solubility of small-molecule quinone, thereby enhancing the cycle stability of the battery.

**Fig. 1:**
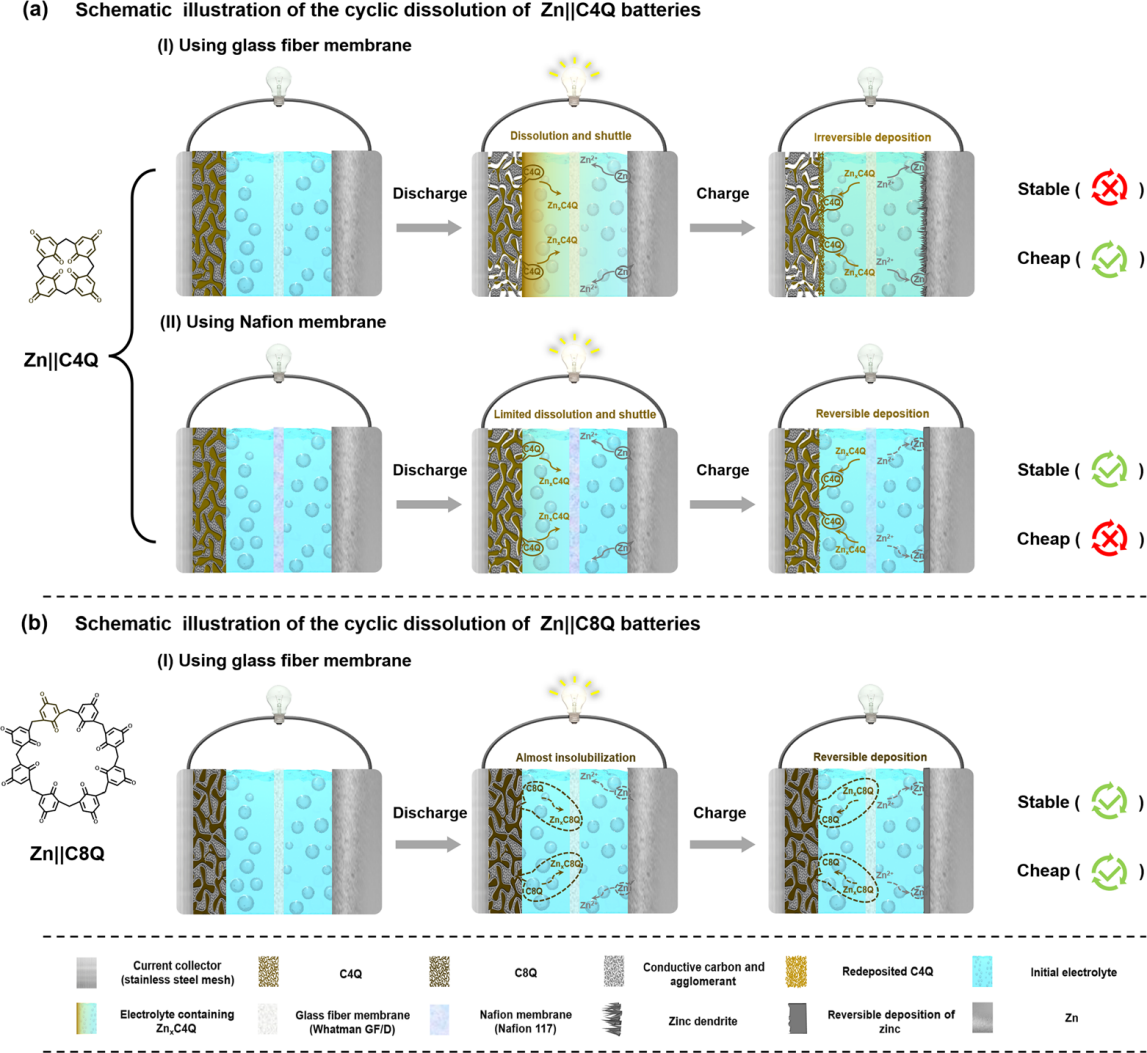
Schematic of the cyclic dissolution of (a) Zn‖C4Q and (b) Zn‖C8Q batteries.

Building upon the aforementioned dissolution experiment analysis, a schematic illustration of the cyclic dissolution of C4Q and C8Q cathodes in 3.0 M Zn(OTf)_2_ electrolyte is presented (Fig. 1). Benefiting from the almost insoluble characteristics of C8Q and ZnxC8Q in aqueous electrolytes, the Zn‖C8Q battery achieved dual-effect optimization of the cost and electrochemical stability of AZIBs. The rationalized molecular design strategy for C8Q has a positive guiding significance to solving the dissolution problem of organic quinone cathodes.

### Electrochemical performances of the C8Q cathode in AZIBs

In AZIBs, the cathodes with outstanding electrochemical properties would typically impart AZIBs with high capacity and rate performance, while the stability of the zinc anode is the key to determining whether AZIBs can achieve a stable long cycle. Several studies have proven that the concentration of electrolytes usually has a great effect on the composition of the solvated shell of Zn^2+^ in AZIBs, which then affects the uniformity of zinc deposition on the zinc anode side.^35,36^ Hence, to construct stable Zn‖C8Q batteries, we selected 3.0 M Zn(OTf)_2_ electrolyte for an in-depth analysis of characterization and testing in the subsequent experiments (the detailed screening and comparison can be seen in Fig. S9 in the ESI†).

**Fig. 2:**
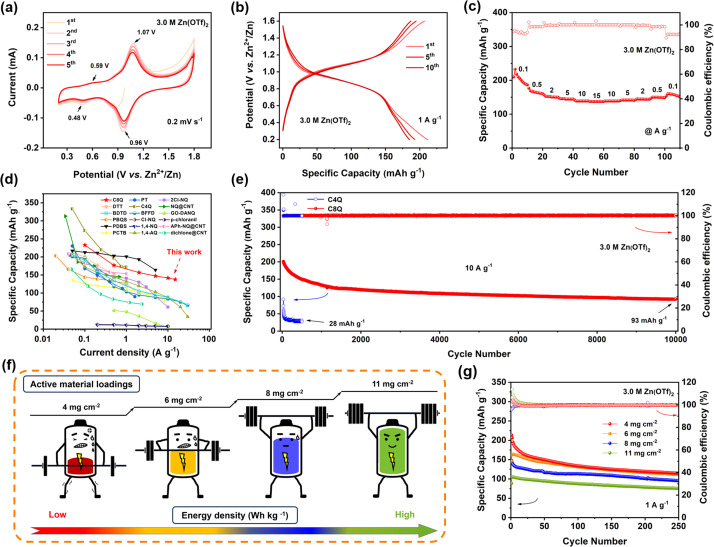
The electrochemical performance of the C8Q cathode in 3.0 M Zn(OTf)_2_: (a) CV curves at 0.2 mV s^−1^. (b) Discharge/charge curves at 1 A g^−1^. (c) Rate capability. (d) Rate capability of the Zn‖C8Q battery compared with other aqueous Zn-organic batteries. (e) Ultra-long cycle performances of the C4Q cathode and C8Q cathode under 10 A g^−1^. (f) Schematic of Zn‖C8Q batteries under high active material loadings. (g) Cycle performance of the C8Q cathodes with the active material loadings of 4, 6, 8, and 11 mg cm^−2^ at 1 A g^−1^.

As illustrated in Fig. 2a, the cyclic voltammetry (CV) test was implemented under the scan rate of 0.2 mV s^−1^. It could be observed that the C8Q cathode possessed two pairs of redox peaks with different intensities in 3.0 M Zn(OTf)_2_. Among them, a pair of weaker redox peaks were observed around 0.48/0.58 V, while a stronger pair was located around 0.96/1.07 V. Notably, the subsequent multiple cycles maintained a high level of consistency in the CV curves, revealing small polarization and splendid redox reversibility, whereafter the C8Q cathode was examined by constant current cycling and variable current cycling tests. At 1 A g^−1^, the initial discharge-specific capacity of the C8Q cathode was 207.2 mA h g^−1^. Over the subsequent 250 cycles, the discharge-specific capacity of the C8Q cathode gradually stabilized with the average coulombic efficiency near 100% (Fig. 2b and S10†).

Fig. 2c and S11a† show the rate performance of C8Q at various current densities. At 0.1, 0.5, 2, 5, 10, and 15 A g^−1^, the reversible discharge-specific capacity of the C8Q cathode reached 232.8, 176.6, 156.8, 147.3, 141.7, and 137.5 mA h g^−1^, respectively. As depicted in Fig. 2d, its rate performance surpasses that of most previously reported similar organic cathodes with *p*-benzoquinone unit structures for AZIBs. As the current density gradually decreased from 15 A g^−1^ to 0.1 A g^−1^, the specific discharge capacity of the Zn‖C8Q battery would increase correspondingly, indicating that the C8Q cathode showed superior rate performance. Due to the good operating voltage and distinguished rate performance, the C8Q cathode could deliver a high specific energy density of 189 W h kg^−1^ at 80 W kg^−1^. Even at 11 623 W kg^−1^, its energy density could still remain at 107 W h kg^−1^ (Fig. S11b†). Remarkably, the Zn‖C8Q battery assembled with 3.0 M Zn(OTf)_2_ also displayed terrific cycle stability under the high current density of 10 A g^−1^. After 10 000 ultra-long cycles, the C8Q cathode still showed a discharge capacity of 93 mA h g^−1^ (0.0059% capacity decay/cycle) (Fig. 2e). In sharp contrast, after only 500 cycles at 10 A g^−1^, the discharge-specific capacity of the Zn‖C4Q battery rapidly decayed to 28 mA h g^−1^.

Based on the outstanding cycle stability of C8Q in aqueous electrolytes, and to pursue high energy density batteries at the same time, we further explored the electrochemical performance of the C8Q cathodes under different high-mass loadings (Fig. 2f). As depicted in Fig. 2g and S12,† after 250 cycles at 1 A g^−1^, the discharge-specific capacity of the C8Q cathodes with four different active material loadings gradually stabilized. The recorded discharge-specific capacities were as follows: 110.9 mA h g^−1^ (4 mg cm^−2^), 107.9 mA h g^−1^ (6 mg cm^−2^), 95.3 mA h g^−1^ (8 mg cm^−2^), and 75.6 mA h g^−1^ (11 mg cm^−2^), respectively. These values correspond to 86.8%, 84.4%, 74.6%, and 59.2% of the capacity (127.8 mA h g^−1^) achieved at 1 mg cm^−2^. Besides, the C8Q cathodes also suggested splendid rate performance at different active material loadings with variable current density, ranging from 0.25 A g^−1^ to 4 A g^−1^ (Fig. S13 and S14†). For instance, at 8 mg cm^−2^, the C8Q cathode displayed a capacity of 103.2 mA h g^−1^ at 4 A g^−1^, retaining 72.7% of that achieved at 0.25 A g^−1^ (142.0 mA h g^−1^). It is worth noting that, as the active material loadings were increased, the Zn‖C8Q batteries experienced a greater decline in capacity. Additionally, this capacity decay of AZIBs was further aggravated at a high current density. This occurrence was ascribed to the elevated mass loading, thereby leading to a much higher areal current density.^37^ Simultaneously, as the active material loading of C8Q cathodes increased, the limited electrode interface failed to achieve rapid interfacial charge transfer, leading to the decrease of its actual discharge-specific capacity. Nonetheless, the C8Q cathodes at active material loadings of 4, 6, and 8 mg cm^−2^ all maintained excellent cyclic stability in the large-current long-cycle test of 4 A g^−1^, with an average CE of 99.8% (Fig. S15†). Especially at an active material loading of 11 mg cm^−2^, the C8Q cathode could still achieve a stable and reversible charge–discharge cycle. Regrettably, the stability of the zinc anode was also greatly challenged when the C8Q cathode was under high active material loadings. In the actual test process, the growth of zinc dendrites was more serious under high active material loadings, which eventually led to a decrease in the overall battery cycle stability. In the follow-up research, while focusing on the development of high-performance organic cathodes, we will also explore some related research on the stability of the zinc anode.

### Reaction kinetics

**Fig. 3:**
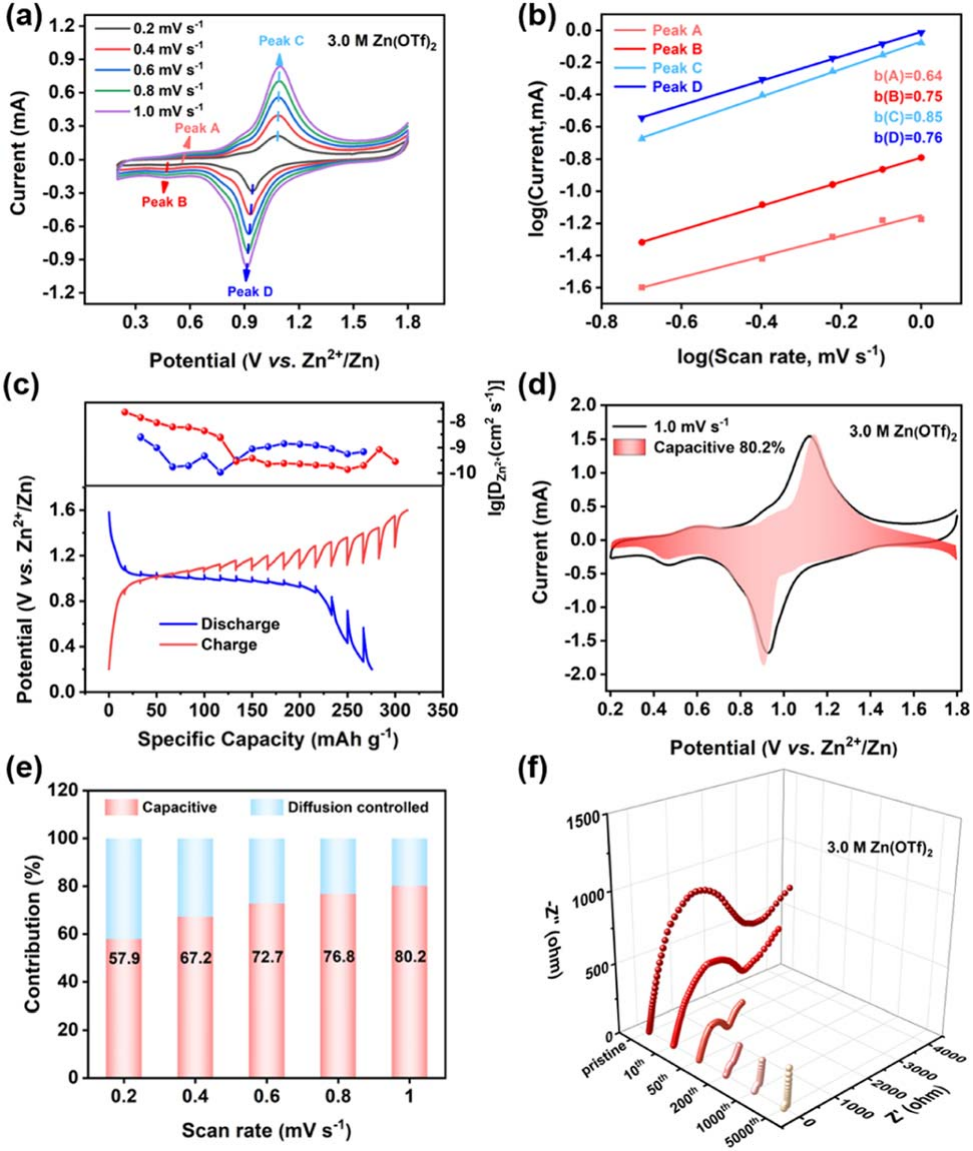
(a) CV curves of the C8Q cathode at different scan rates. (b) Logarithmic relationship of the C8Q cathode between the peak currents and the scan rates. (c) *D*_Zn^2+^_ of AZIBs with the C8Q cathode at 50 mA h g^−1^. (d) Contribution of the capacitance control at 1.0 mV s^−1^. (e) Capacity contribution of nondiffusive control and diffusion control at different scan rates. (f) EIS plots of the C8Q cathode before cycling (pristine) and after the 10th, 50th, 200th, 1000th, and 5000th cycles at 0.5 A g^−1^.

To elucidate the origin of its extraordinary electrochemical performance, the reaction kinetics of C8Q was investigated. First, CV was conducted at different scan rates ranging from 0.2 to 1.0 mV s^−1^. With the escalation of the scan rate (Fig. 3a), the peak shape of the redox peaks in the CV curve remained highly consistent with only slight polarization, demonstrating the remarkable redox reversibility of C8Q. Then, the linear relationship between peak current (log *i*) and scan rate (log *v*) was established using eqn (1) and (2) in the ESI.† The fitted *b* values for the four redox peaks were determined to be 0.64, 0.75, 0.85, and 0.76, respectively (Fig. 3b). The determined *b* values, consistently approximating 0.75 (between 0.5 and 1.0), imply that the charge storage mechanism of C8Q is regulated by a concurrent interplay of diffusion and capacitance processes,^38^ whereafter the effects of the diffusion and capacitance control were further evaluated.

The ion diffusion coefficient of Zn^2+^ (*D*_Zn^2+^_) was determined through the application of the galvanostatic intermittent titration technique (GITT) (eqn (3) in the ESI†). In Fig. 3c, the diffusion coefficient *D*_Zn^2+^_ of the C8Q cathode ranged from 10^−10.5^ to 10^−7.5^ cm^2^ s^−1^, which was relatively superior in aqueous zinc-organic batteries.^39^ Commonly, the capacitance behavior could deliver rapid interfacial charge transfer kinetics, thereby bolstering the rate performance of batteries.^40^ Consequently, the proportions between the contribution of diffusion and capacitance were further computed (eqn (3) in the ESI†). Fig. 3d and e show the value of pseudocapacitance contribution of the C8Q cathode was 57.9–80.2% at 0.2 to 1.0 mV s^−1^, implying the predominant role of interfacial charge transfer in energy storage. With the increasing scanning rate, the value of capacitance contribution rose and the influence of interfacial charge transfer control was further strengthened. The enhanced capacitance attribute of the C8Q cathode gives it the rapid charge transfer ability as the current density increases, making the capacity decay smaller at a large current density (5 A g^−1^ to 15 A g^−1^) (Fig. 2c). Notably, during the cycle, the interfacial charge transfer resistance (*R*_ct_) of the C8Q cathode decreased rapidly. The swift reduction in *R*_ct_ facilitated fast interfacial charge conduction and accelerated the redox reaction, contributing to the long-period stability of the C8Q cathode (Fig. 3f). Clearly, the above kinetic studies suggested a close connection between interfacial charge transfer and the electrochemical performance properties of C8Q cathodes.

### Energy storage mechanism of C8Q

**Fig. 4:**
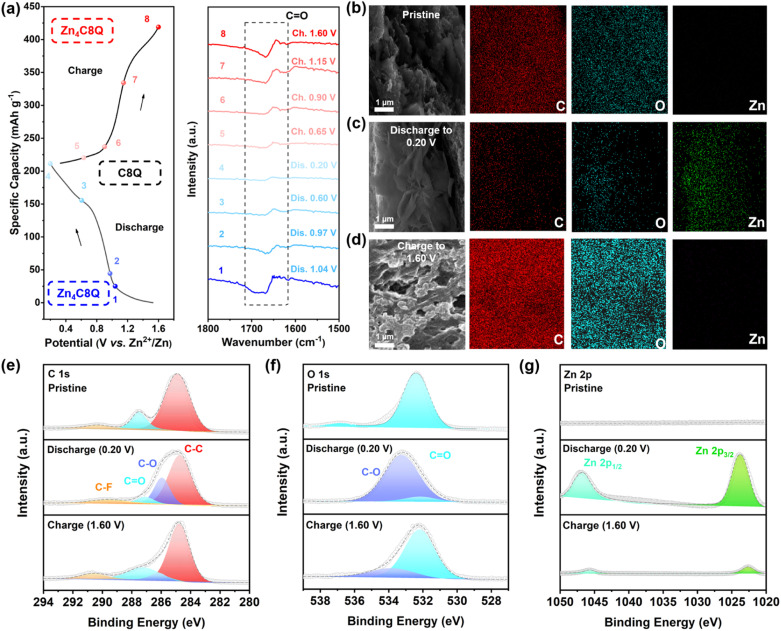
(a) GCD curve of the C8Q-based AZIBs at 1 A g^−1^ and the corresponding *ex situ* FT-IR spectra. The SEM-EDS images of the C8Q cathode at different states: (b) pristine, (c) discharged to 0.20 V, and (d) recharged to 1.60 V. (e) C 1s, (f) O 1s and (g) Zn 2p XPS spectra of C8Q cathodes across pristine, fully discharged, and fully charged states.

In general, the electrochemical reaction kinetics is one of the fundamental causes for the differences in the performance of the electrode materials, while different charge storage mechanisms might determine the speed of the electrochemical reaction kinetics. Thus, our attention was directed toward a meticulous investigation of the nuanced charge storage mechanism exhibited by C8Q in AZIBs. The galvanostatic charge–discharge (GCD) curves and the corresponding *ex situ* FT-IR spectra are illustrated in Fig. 4a. Throughout the discharge from 1 (1.04 V) to 4 (0.20 V), the vibrational peak corresponding to C=O gradually disappeared; while charging from 4 (0.20 V) to 8 (1.60 V), the vibration peak of C=O gradually increased, indicating the reversible conversion of C=O. Furthermore, the X-ray photoelectron spectroscopy (XPS) analysis of C8Q revealed more details on the composition transformation including C, O, and Zn elements (Fig. 4e–g and S16†). In the C 1s spectrum (Fig. 4e), the intensity of the C=O peak at 284.7 eV exhibited a gradual reduction when fully discharged (0.20 V).^41^ In the meantime, a new peak of C–O emerged, with its intensity progressively increasing. Conversely, the exact opposite changes were observed when fully charged (1.60 V). From the O 1s spectrum (Fig. 4f), the intensities of C=O and C–O peaks also displayed similar trends to those in the C 1s spectrum during the charge–discharge process, again corroborating the reversible transition between C=O and C–O.^42^ Moreover, the Zn 2p XPS spectrum (Fig. 4g) showed that two distinct split peaks, specifically Zn 2p_1/2_ and Zn 2p_3/2_, were characterized by a substantial intensity of the zinc element during the full discharge state.^43^ However, these two split peaks associated with the Zn element virtually dissipated upon recharging to 1.60 V, signifying the reversibility of the zinc-ion insertion/extraction.

The SEM-EDS profile under TEM observation displayed no significant alteration in the distribution of C and O elements in the pristine, fully discharged, and fully charged C8Q cathodes (Fig. 4b–d). However, the changing trend of the Zn element remained consistent with the Zn 2p XPS spectrum described above, displaying a reversible increase and decrease. Notably, the micromorphology of the C8Q cathode was greatly changed under different charge and discharge states. The XRD analysis further demonstrated the transition of the crystal structure of the C8Q molecule during the intercalation and deintercalation events of Zn^2+^ (Fig. S17†). In the discharge state, the surface of the C8Q cathode predominantly exhibited a relatively smooth sheet layer structure. Nevertheless, in the initial state and the charging state, the electrode surface presented a nanosized multidimensional porous channel structure. These reversible structural changes greatly augmented the charge transfer interface area of the electrode, enhanced the accessibility of the electrolyte, and facilitated the rapid conduction of the interfacial charge. This, in turn, led to rapid reaction kinetics dominated by interfacial load transfer control, contributing to good electrochemical performance of C8Q at the high current density mentioned above.

Due to the hydrolysis reaction of Zn^2+^, a certain amount of free protons (H^+^) is typically generated in the aqueous solution of ZnSO_4_, Zn(OTf)_2_, or ZnCl_2_, resulting in the pH of the electrolyte being 4–5. Although some previous reports have verified that carbonyl-based organic cathodes can simultaneously combine with H^+^ and Zn^2+^ during the discharge process, this phenomenon does not seem to be universally applicable to all carbon-based organic cathode materials. To ascertain whether H^+^ is involved in the storage, the typical three-electrode method was employed to test the CV curves of C8Q cathodes across three distinct electrolytes: 3.0 M Zn(OTf)_2_/H_2_O electrolyte (pH = 4.35), CF_3_SO_3_H/H_2_O electrolyte (pH = 4.35), and saturated Zn(OTf)_2_/acetonitrile (AN) electrolyte. In 3.0 M Zn(OTf)_2_/H_2_O electrolyte containing both Zn^2+^ and H^+^, two pairs of redox peaks (I, II, III, IV) appeared, and the shape of the peaks highly matched the CV curve under the coin-cell system. When containing only H^+^ in CF_3_SO_3_H/H_2_O electrolyte (Fig. S18a†), discernible redox peaks for the C8Q cathode were absent. Additionally, the CV curve in CF_3_SO_3_H/H_2_O electrolyte differed greatly from that in 3.0 M Zn(OTf)_2_/H_2_O electrolyte. In contrast, when containing only Zn^2+^ in saturated Zn(OTf)_2_/AN electrolyte, the C8Q cathode displayed four distinct redox peaks (I′, II′, III′, IV′), which correspond to the four redox peaks (I, II, III, IV) observed in the 3.0 M Zn(OTf)_2_/H_2_O electrolyte (Fig. S18b†).

Remarkably, the shape and relative intensity of the redox peaks in the two CV curves in Fig. S19b† also corresponded one to one, indicating the absence of cooperative participation of H^+^ in the C8Q cathode in 3.0 M Zn(OTf)_2_/H_2_O electrolyte.

The above results confirmed that Zn^2+^ underwent reversible redox reactions with C=O of C8Q, realizing energy storage and conversion. The C8Q cathode exhibited a reversible specific capacity of 232.8 mA h g^−1^ at 0.1 A g^−1^, corresponding to the redox process of 8 electrons. Therefore, C8Q could be reversible to combine/separate with four Zn^2+^ for energy storage and transformation.

### Flexible electronic device applications

**Fig. 5:**
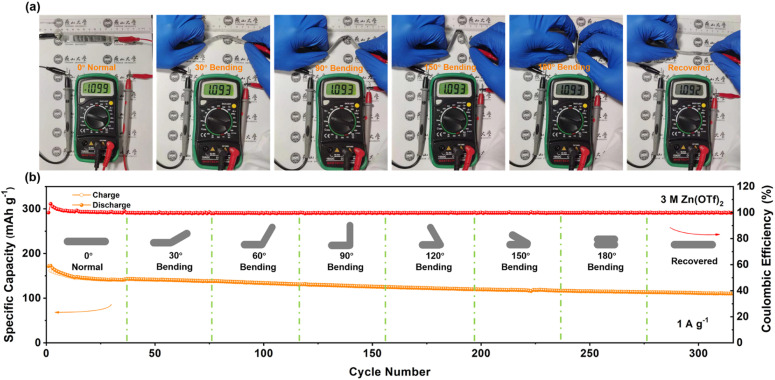
(a) Bending test of the belt-shaped battery at different angles. (b) Cycle stability through consecutive and repeated bending states at 1 A g^−1^.

Given the exceptional cycle stability exhibited by the C8Q cathode in coin batteries, we proceeded to assemble a belt-shaped Zn‖C8Q battery to try its application in flexible electronic devices (Fig. S19 and S20a†). As depicted in Fig. 5a, the output voltage of the belt-shaped battery remained stable under various bending states (normal, 30°, 90°, 150°, 180°, and recovered). Moreover, in other bending cases, the belt-shaped battery also exhibited excellent cycle stability at 0.5 A g^−1^ and 1 A g^−1^, indicating robust bending resistance and stable mechanical performance (Fig. 5b and S20c†). In Fig. S20b,† two newly assembled belt-shaped batteries connected in series could provide a stable output voltage of 2.19 V, successfully driving and illuminating an LED bulb. This demonstrates the broad prospects for the application of Zn‖C8Q batteries in the domain of flexible and wearable electronic devices.

## Conclusions

In summary, a C8Q molecule containing *p*-benzoquinone structural units was for the first time proposed as a viable cathode material for AZIBs. Distinct from the classical small-molecule quinone C4Q, the C8Q cathode realized the high solubility limit of C8Q and its discharge products in aqueous electrolytes. In 3.0 M Zn(OTf)_2_, the C8Q cathode demonstrated a remarkably reversible capacity of 207.2 mA h g^−1^ at 1 A g^−1^, prolonged cycle stability over 10 000 cycles (0.0059% capacity decay per cycle), and satisfactory rate performance. Even under an active material loading of 11 mg cm^−2^, the C8Q cathode also maintained stable and highly reversible electrochemical zinc storage in AZIBs. Electrochemical kinetic studies elucidated that the exceptional electrochemical performance of the C8Q cathode originated from the rapid interfacial charge transfer ability conferred by the pseudo-capacitive properties. Furthermore, *ex situ* characterizations and electrochemical measurements unveiled that the C8Q cathode underwent the 4Zn^2+^ reaction with the carbonyl groups in the C8Q molecules during the discharge/charge process. It is particularly noteworthy that a belt-shaped Zn‖C8Q battery displayed excellent flexibility. We believe that this research, based on the rational design of redox organic cathodes, provides guidance for the construction of the next generation of sustainable AZIBs. Furthermore, with the continuous optimization of the AZIB system, AZIBs will shine in practical fields of flexible electronics and large-scale energy storage.

## Data availability

All experimental supporting data and procedures are available in the ESI.†

## Author contributions

Q. C. Zhang and W. W. Huang directed and supervised the project. C. J. Ding carried out the experiment, materials characterization, data analysis and writing – original draft. Y. H. Wang and C. B. Li contributed to the experiment, data analysis and writing – review & editing. J. W. Wang contributed to writing – review & editing.

## Conflicts of interest

The authors declare no conflict of interest.

## Supplementary Material

SC-015-D4SC00491D-s001
