# Supplementary material for: Constructing ultra-stable, high-energy, and flexible aqueous zinc-ion batteries using environment-friendly organic cathodes
Source: Chem Sci. 2024 Feb 27;15(13):4952–9. doi: 10.1039/d4sc00491d (PMC10967254; doi:10.1039/d4sc00491d)
Supplement: SC-015-D4SC00491D-s001 [file SC-015-D4SC00491D-s001.pdf]

Electronic Supplementary Material (ESI) for Chemical Science.
This journal is © The Royal Society of Chemistry 2024

Electronic Supplementary Information

Constructing ultra-stable, high-energy, and flexible aqueous zinc-ion batteries using environment-friendly organic cathodes

Chaojian Ding,[a] Yonghui Wang,[a] Chaobo Li,[a] Jiawen Wang,[a] Qichun Zhang[*bc] and Weiwei Huang[*a]

[a] *Hebei Key Laboratory of Applied Chemistry, Yanshan University, Qinhuangdao 066004, Hebei, China.*

[b] *Department of Materials Science and Engineering, City University of Hong Kong, Hong Kong SAR 999077, China.*

[c] *Center of Super-Diamond and Advanced Films (COSDAF) & Department of Chemistry, City University of Hong Kong, Hong Kong SAR 999077, China.*

* Corresponding authors: qiczhang@cityu.edu.hk (Q. Zhang), huangweiwei@ysu.edu.cn (W. Huang).

Experimental Section

Materials

Potassium hydroxide (KOH, AR), xylene (Xyl, AR), acetone (DMK, AR), petroleum ether (PE, AR), trichloromethane (TCM, AR), methanol (MeOH, AR), and N-methyl-2-pyrrolidone (NMP, 99.9%) Sodium hydrosulfite ($Na_2S_2O_4$, 90%), dimethyl sulfoxide (DMSO, AR), acetic acid (HAc, AR), ethyl acetate (EA, AR), ethanol (EtOH, AR), hydrochloric acid (HCl, 36%), sulphuric acid (H_2SO_4, 98%), 4-benzyl oxyphenol (PBP, 98%), paraformaldehyde (POM, AR), iodotrimethylsilane (TMSI, 97%), iron(III) chloride anhydrous ($FeCl_3$, AR), and potassium dichromate ($K_2Cr_2O_7$, AR) were used as purchased without further purified.

Zinc trifluoromethanesulfonate ($Zn(OTf)_2$, 98%) was applied as the donor of active Zn^{2+}. Polyvinylidene fluoride (PVDF), polytetrafluoroethylene (PTFE, 60%wt), and Ketjen Black (KB) were obtained for preparing the organic composite electrodes. The current collector was soft stainless steel mesh and the separator was the Whatman glass filter membrane (GF/D).

Synthesis of *p*-(Benzyloxy) Calix[8]arene

7.5 g (37.5 mmol) of 4-benzyl oxyphenol, 2.0 g (66.675 mmol) of paraformaldehyde, 0.75g (1.125 mmol) of potassium hydroxide and 90 mL of xylene were slowly added to a four-mouth round bottom flask with oil-water separator. The mixture was refluxed for 6 h and cooled to room temperature. After filtration, a gray-white coarse product was obtained. This product was subsequently rinsed with xylene, acetone, and petroleum ether successively to obtain 3.320 g of a white powdered solid with a yield of 41.8%.

Synthesis of Calix[8]hydroquinone

Remove 2.8g (1.6 mmol) of *p*-(Benzyloxy) Calix[8]arene in a triple flask containing 100 mL chloroform, then slowly add 10 mL of (70 mmol) iodotrimethylsilane. The above mixture was stirred under reflux in a nitrogen atmosphere for 12 h, then cooled to room temperature with 5 mL of methanol. Subsequently, the solvent was removed, yielding a dark solid. The product was suspended in 5 mL of $CHCl_3$ and treated with 100 mL of a 1% solution of $Na_2S_2O_4$. After stirring at room temperature for 2

hours, the separated products were filtered under a nitrogen charge (85.8% yield).

Synthesis of Calix[8]quinone (C8Q)

The 0.37 g (0.382 mmol) of Calix[8]hydroquinone was dissolved in 25 mL of glacial acetic acid heated to 50 °C under a nitrogen atmosphere. Simultaneously, 2.45 g of $FeCl_3$ was added to 10 mL of HCl solution (18%) and stirred thoroughly for 15 minutes until the solution turned yellow-green. The above yellow-green solution was then poured into a solution of 1.47 g of potassium dichromate solution and 3.85 mL of concentrated H_2SO_4 in 75 mL water, followed by heating to 90 °C for 15 minutes. After cooling to room temperature, the solution was filtered to obtain a yellow-green solid product. The resulting crude product was dissolved in DMSO, stirred at room temperature for 30 min, and then extracted and filtered. The filter residue underwent sequential washes with glacial acetic acid, acetone, ethyl acetate, and petroleum ether. The filter was finally vacuum-dried vacuum at 60 °C to give 0.127 g of yellow solid (M=960) with a yield of 62.8%.

Materials characterizations

The composition and structure of Calix[8]quinone were measured by electrospray ionization mass spectrometry (ESI-MS, CQ Finnigan) and nuclear magnetic resonance (NMR, AVANCE III 400, Germany) with trifluoroacetic acid-d (CH_3COOD) as the solvent. Functional groups were characterized by Fourier transform infrared (FTIR, FTIR-650, China). Crystal configuration was performed on the X-ray diffractometer (XRD, Rigaku D/MAX 2500, Japan) with Cu Kα radiation in the wide 2θ range of 5-60° at 5° min^{-1}. The morphologies of the active materials were carried out by scanning electron microscopy (SEM, Zeiss Merlin Compact, Germany). The molecular structure and atomic valence state information of the samples were analyzed by X-ray photoelectron spectrometer (XPS, Thermo Scientific, America).

Electrochemical Measurements

Preparation of C8Q electrodes

40 wt.% active material (C8Q), 40 wt.% conductive additive (KB), and 20 wt.% binders (PVDF) were ground in an agate mortar with NMP as the solvent. The electrode slurry was fully wet-milled until

uniformly and particle-free. Subsequently, the slurry was coated onto the surface of a soft stainless steel mesh, transferred to an oven, and dried at 80 °C for 12 hours. Finally, the soft stainless steel mesh was cut into a 12 mm diameter disc, resulting in an active material loading of 1 mg cm^{-2}.

The high-loading C8Q cathodes (50 wt.% C8Q, 40 wt.% KB carbon, and 10 wt.% PTFE) were prepared with absolute ethanol as the solvent. The mixture was then pressed onto soft stainless steel mesh with the electric roller machine. The mass loading of C8Q was in the range of 4-11 mg cm^{-2}.

Assembly process of aqueous zinc-ion batteries (AZIBs)

The button-type CR2032 half-cells were assembled with C8Q as the cathode, zinc foil (≥99.999%) as the anode, 1.5 M, 2.0 M, 3.0 M $Zn(OTf)_2$ aqueous solution as the electrolytes, and the glass fiber membrane (Whatman GF/D) as the separator with a diameter of 16 mm.

Assembly flexible belt-shaped AZIBs

The flexible belt-shaped battery was fabricated by the following procedure: (i) The prepared C8Q cathode (50 wt.% C8Q, 40 wt.% KB carbon, and 10 wt.% PTFE), the Whatman GF/D membrane, and the zinc foil were cut in a size of 1.0 × 8.5 cm (the actual loading area of cathode materials was 1.0 × 5.5 cm), 1.2 × 6.5 cm, and 1.2 × 6.5 cm, respectively. Then a section of 1.0 × 3.0 cm soft stainless steel mesh was attached to one end of the zinc foil to act as a polar ear. (ii) These components were placed in a long strip of a plastic seal bag and assembled as a sandwich structure. (iii) 1.2 mL of 3.0 M $Zn(OTf)_2$ aqueous solution was added. Finally, a belt-shaped Zn||C8Q battery was obtained by a vacuum-sealed operation. The active material loading of the C8Q cathode was 2.0-2.5 mg cm^{-2}.

Electrochemical characterizations

The cyclic voltammetry (CV) and electrochemical impedance spectroscopy (EIS) of C8Q-based AZIBs were performed on the CHI-660E electrochemical workstation. The galvanostatic charge-discharge (GCD), galvanostatic intermittent titration technique (GITT) tests, and rate performances were conducted on the Neware CT-4008T test system and the LANHE-CT2001A test system. The typical three-electrode test was performed with the C8Q cathode as the working electrode, the Ag/AgCl electrode as the reference electrode, and the graphite electrode as the counter electrode.

SUPPLEMENTARY NOTES AND FIGURES

1. The price comparison between Nafion membrane and glass fiber membrane (Whatman GF/D).

Data sources: The glass fiber membrane (Whatman GF/D) was purchased from the Duoduo reagent network, 2023 (https://www.dodochem.com/); The DuPont Nafion 117 was purchased from Suzhou Sinero Technology Co., Ltd, 2023 (http://sinerosz.com/).

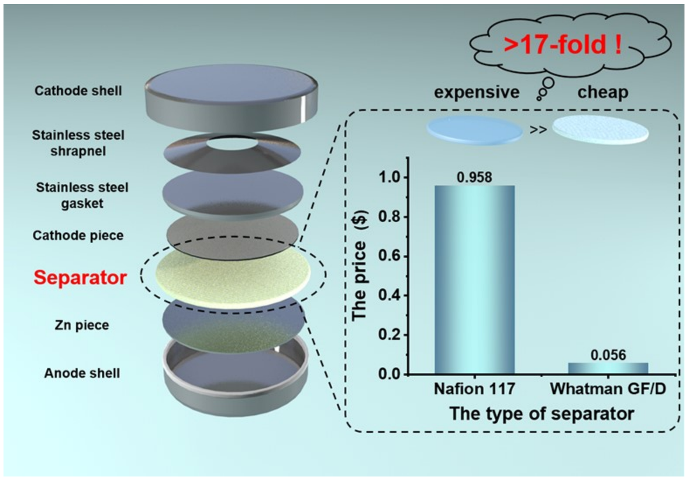

Fig. S1. Price of a piece of Nafion membrane or Whatman glass fiber membrane (D=16 mm) in coin-type (CR2032) batteries.

2. The synthesis of C8Q.

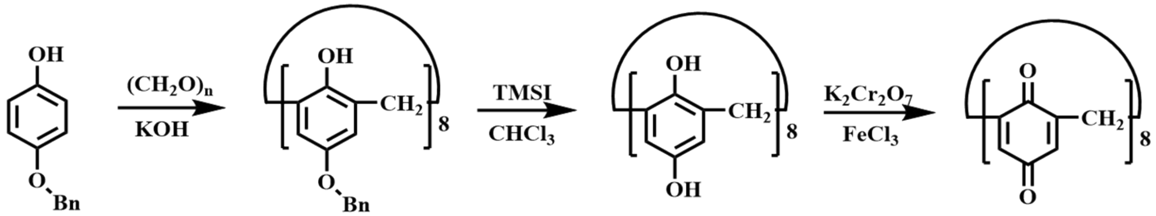

Scheme S1. Synthetic processes of C8Q.

3. The ESI-MS of C8Q.

There was a distinct fragment peak (m/z=959.20), corresponding to the M-1 fragment peak of C8Q (m/z=960.17).

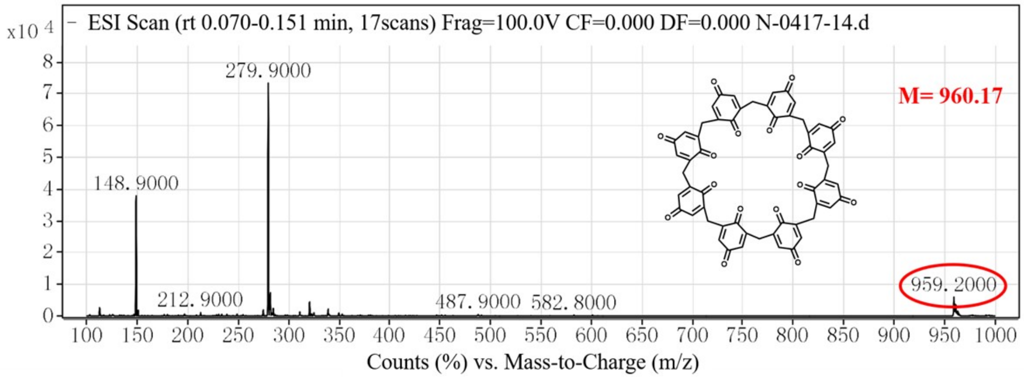

Fig. S2. ESI-MS spectra of C8Q.

4. The ^{1}H NMR spectra of C8Q.

As observed from the ^{1}H NMR spectrum of C8Q (in CF_3COOD) (Fig. S2), significant peaks appeared at δ of 11.55, 6.95, and 3.79, corresponding to the characteristic structures of CF_3COOD and C8Q (s, 16H, -CH=C-; s, 16H, $-CH_2-$), respectively.

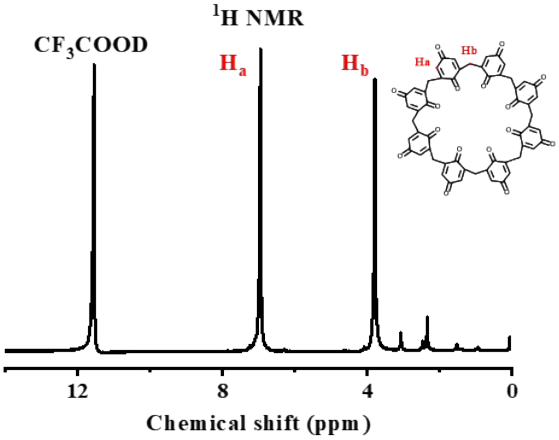

Fig. S3. ^{1}H NMR spectra of C8Q with CF_3COOD as the solvent.

5. The structural and morphological characterizations of C8Q.

In the FTIR spectrum of C8Q (Fig. S4a), the absorption peak at 1649 cm^{-1} was caused by the tensile vibration of the carbon group (C=O) on the p-benzoquinone unit. Additionally, the peak at 1297 cm^{-1} was the bending vibration absorption peak, corresponding to the methylene group ($-CH_2-$) connecting the benzoquinone unit. The SEM images revealed a regular geometry and a spindle particle shape for C8Q (Fig. S4b, S4c). This morphology was further supported by the XRD spectrum (Fig. S16c), which displayed distinct diffraction peaks, confirming the specific crystalline structure of C8Q.

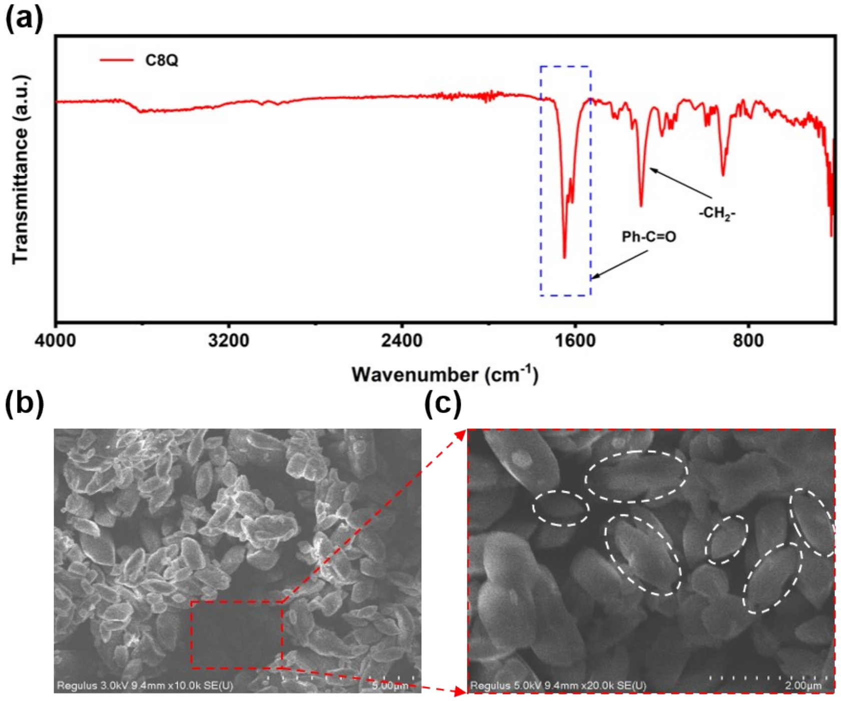

Fig. S4. (a) FTIR spectrum. (b) and (c) SEM image of C8Q.

6. The digital photos of each component of the Zn||C4Q and Zn||C8Q batteries after cycling in 3.0 M $Zn(OTf)_2$.

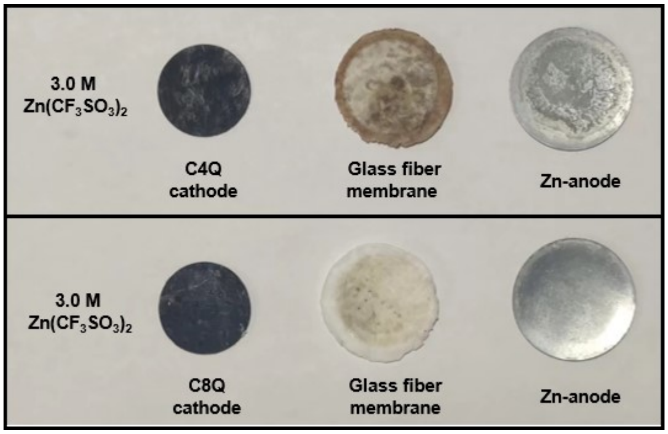

Fig. S5. Digital photos of the Zn anode, separator (glass fiber membrane, Whatman GF/D), C4Q, and C8Q cathode in 3.0 M $Zn(OTf)_2$ after 1000 cycles at 5 A g^{-1}.

7. The static dissolution experiments of the C4Q cathodes.

The C4Q cathode (pristine) and C4Q cathode (discharge to 0.20 V) were added to glass bottles respectively. Subsequently, 2 mL of 3.0 M $Zn(OTf)_2$ electrolyte was added to each bottle, and the solution was sealed for observation. A parallel control group with the only blank electrolyte was established for comparative analysis. The dissolution of C4Q cathodes in the electrolyte was monitored and recorded. On the second, third, fifth, seventh, and fourteenth days, 50 μL of electrolyte was monitored and diluted 40-fold with deionized water. Subsequently, UV spectroscopy was performed on each diluted solution. The results are presented in Fig. S6 below.

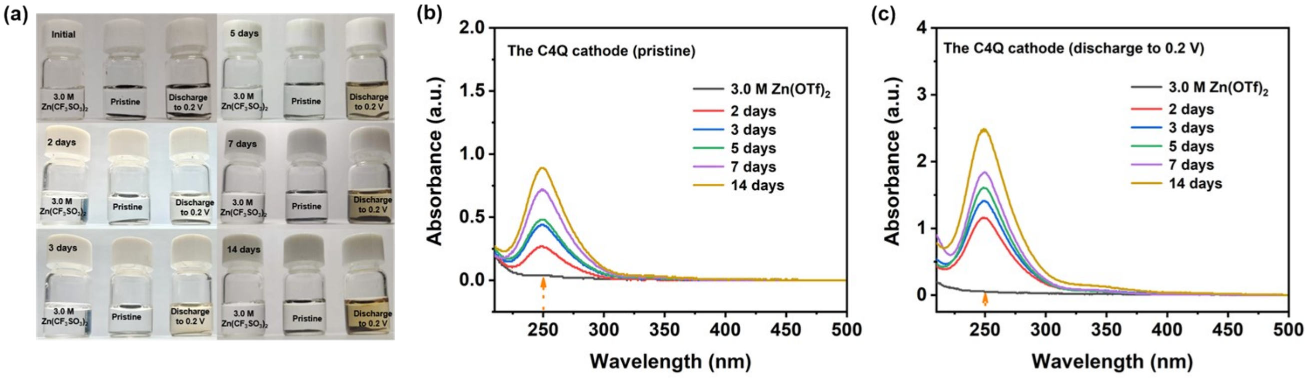

Fig. S6. (a) Static dissolution experiments. UV-vis spectra of (b) the C4Q cathode (pristine) and (c) the C4Q cathode (discharge to 0.20 V) soaked in 3.0 M $Zn(OTf)_2$ for initial, 2 days, 3 days, 5 days, 7 days, and 14 days.

8. The static dissolution experiments of the C8Q cathodes.

The static dissolution experiment of the C8Q cathodes followed a similar operational procedure as the C4Q cathodes. The results are presented in Fig. S7 below.

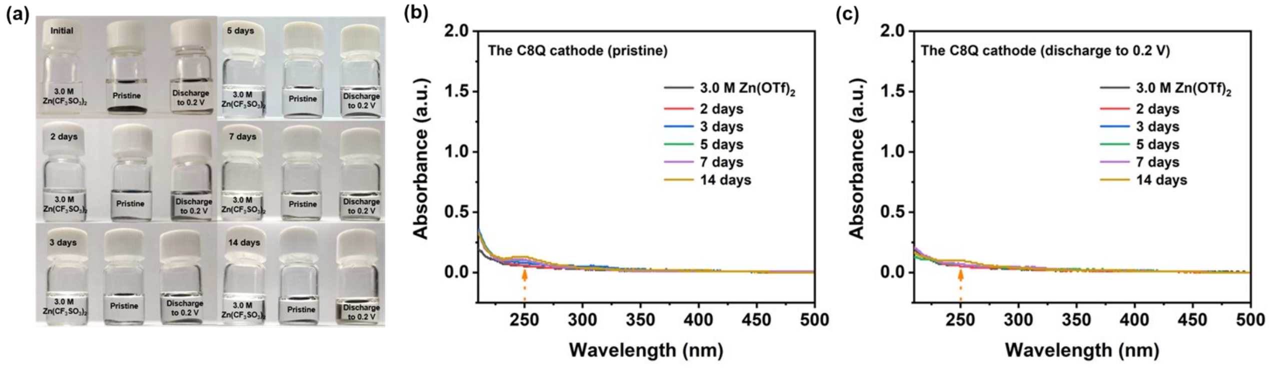

Fig. S7. (a) Static dissolution experiments. UV-vis spectra of (b) the C8Q cathode (pristine) and (c) the C8Q cathode (discharge to 0.20 V) soaked in 3.0 M $Zn(OTf)_2$ for initial, 2 days, 3 days, 5 days, 7 days, and 14 days.

9. The in-situ dynamic dissolution experiments of the Zn||C4Q and Zn||C8Q batteries with 3.0 M $Zn(OTf)_2$ during charge and discharge process.

Initially, the Zn foil, the C4Q cathode, and the C8Q cathode were cut into 0.8×6.0 cm strips (the load area of the electrode material was 0.8×2.0 cm) and placed in the quartz cutlery. Subsequently, a single Whatman GF/D separator was then placed at the bottom of the quartz cutlery to ensure the separation of the cathode and anode, preventing short-circuits. The separator also acted as a white background for the electrolyte. 3 mL of 3.0 M $Zn(OTf)_2$ electrolyte was added into the quartz cutlery, sealed with parafilm membrane. The assembled Zn||C4Q and Zn||C8Q batteries underwent galvanostatic charge-discharge testing at a current density of 0.5 A g^{-1}. Observe and record the dissolution of the C4Q and C8Q cathode during the charge and discharge. One week later, 50 μL of the electrolyte solution in the quartz cutlery was aspirated and diluted 40-fold with deionized water. Then, UV spectroscopy was performed for each diluted solution. On the second, third, fifth, seventh, and fourteenth days, 50 μL of electrolyte was aspirated and diluted 40-fold with deionized water, respectively. Then, UV spectroscopy was performed for each diluted solution.

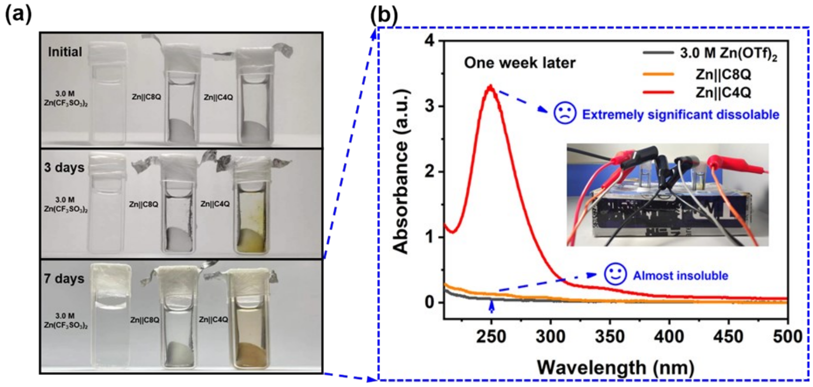

Fig. S8. (a) In-situ dynamic charge-discharge dissolution experiments of the Zn||C4Q battery and the Zn||C8Q battery with 3.0 M $Zn(OTf)_2$ for initial, 3 days, and 7 days. (b) UV-vis spectra of blank, the Zn||C4Q battery and the Zn||C8Q battery with 3.0 M $Zn(OTf)_2$ after one week.

10. The SEM images of the zinc anodes before cycling (pristine) and after cycling in 1.5 M, 2.0 M, and 3.0 M $Zn(OTf)_2$.

As illustrated in Fig. S8a, 8c, and 8e, the SEM deposition behavior analysis of the Zn anode was observed at 1.5 M, 2.0 M, and 3.0 M $Zn(OTf)_2$, respectively. After a single cycle at the current density of 5 A g^{-1}, the uniformity of zinc deposition on the surface of the zinc anode in the low-concentration electrolyte was even worse, accompanied by a small amount of dendrite formation, and the formation of cracks and holes of different sizes. With the increase of the cycling number, the cracks and holes on the surface of the zinc anode in the low-concentration electrolyte were further expanded, showing an obvious "gully" shape. In contrast, the zinc deposition on the surface of the zinc anode was uniformly dense in 3.0 M $Zn(OTf)_2$, without evident sites of zinc dendrite growth (Fig. S8d). Even after 500 cycles, the surface of the zinc anode remained relatively smooth, and the uniformity and reversibility of zinc deposition were significantly better than the former (Fig. S8f).

This observation underscores the positive impact of a higher concentration of $Zn(OTf)_2$ electrolyte in maintaining the stability of the zinc anode while reconstructing the solvated shell of Zn^{2+}.[1,2] Given the heightened stability demonstrated by the Zn anode in the high concentration of $Zn(OTf)_2$ electrolyte, 3.0 M $Zn(OTf)_2$ electrolyte was used for an in-depth analysis of characterization and testing in the subsequent experiments.

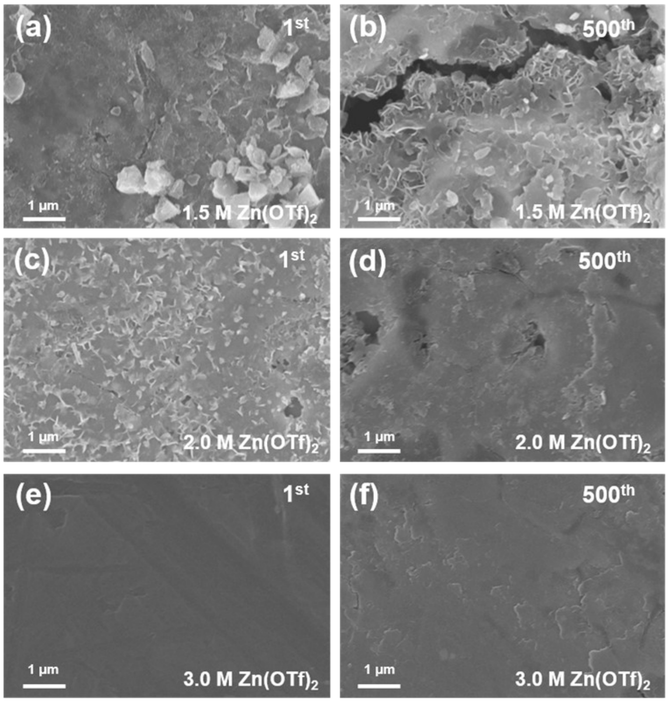

Fig. S9. SEM images of the zinc anodes before cycling (pristine) and after 1 and 500 cycles in (a), (b) 1.5 M, (c), (d) 2.0 M, and (e), (f) 3.0 M $Zn(OTf)_2$.

11. The cycle performance of the C8Q cathode in 3.0 M $Zn(OTf)_2$ at 1 A g^{-1}.

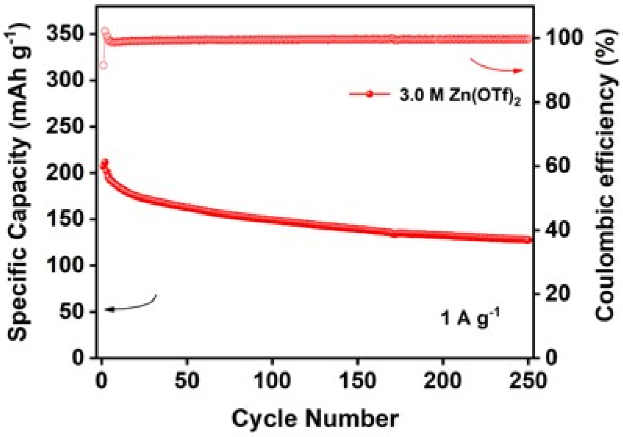

Fig. S10. Cycle performance of the C8Q cathode at 1A g^{-1}.

12. The GCD curves of the C8Q cathode and energy density versus power density in 3.0 M $Zn(OTf)_2$ at various current densities.

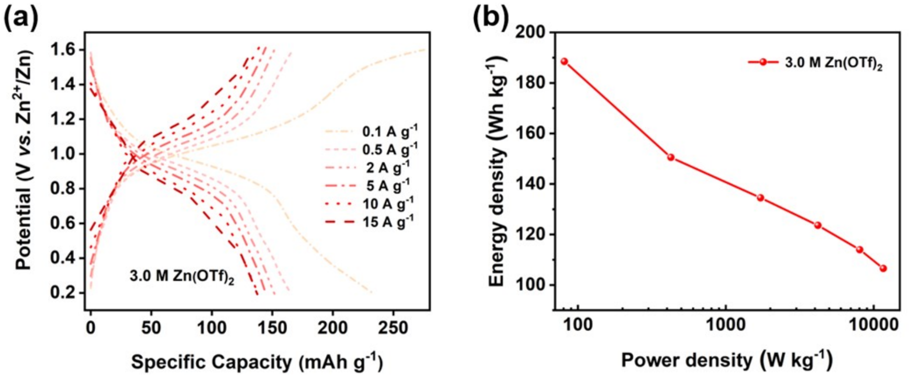

Fig. S11. (a) GCD curves of the C8Q cathode in 3.0 M $Zn(OTf)_2$ at various current densities. (b) Energy density versus power density derived from (a).

13. The GCD curves of Zn||C8Q batteries with different active material loadings in 3.0 M $Zn(OTf)_2$ at the current density of 1 A g^{-1}.

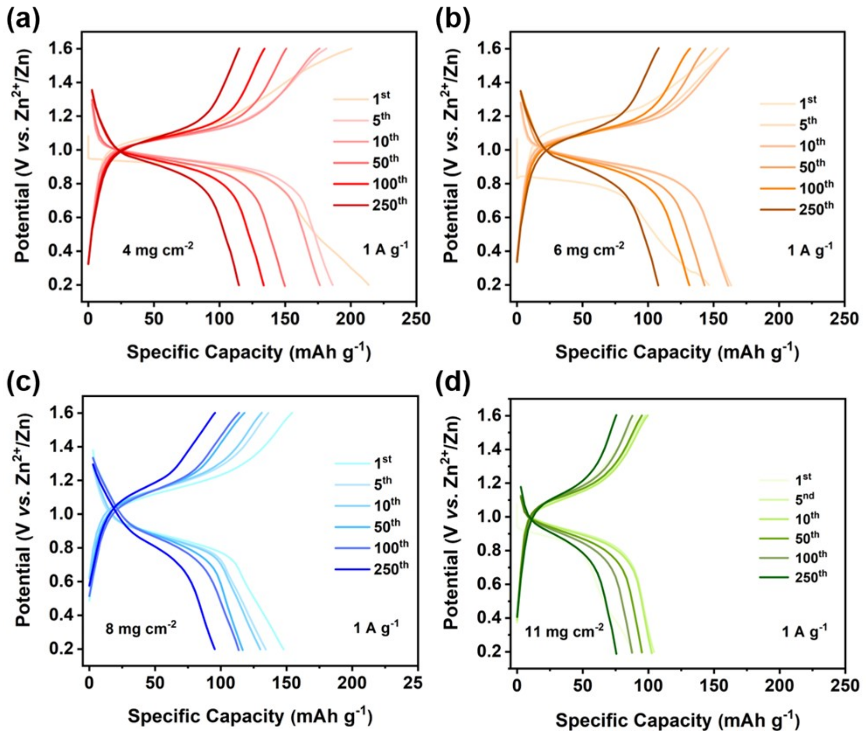

Fig. S12. GCD curves of C8Q cathodes with different active material loadings of (a) 4, (b) 6, (c) 8, and (d) 11 mg cm^{-2} in 3.0 M $Zn(OTf)_2$ at 1 A g^{-1}.

14. The rate capability of the C8Q cathodes with the active material loadings of 4, 6, 8, and 11 mg cm^{-2} in 3.0 M Zn(OTf)$_2$.

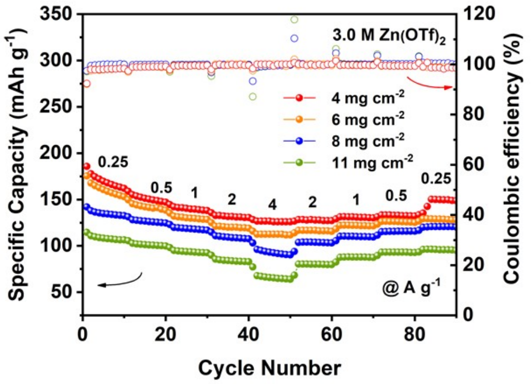

Fig. S13. Rate capability of the C8Q cathodes with the active material loadings of 4, 6, 8, and 11 mg cm^{-2} in 3.0 M Zn(OTf)$_2$.

15. The GCD curves of Zn||C8Q batteries with different active material loadings in 3.0 M Zn(OTf)$_2$ at various current densities.

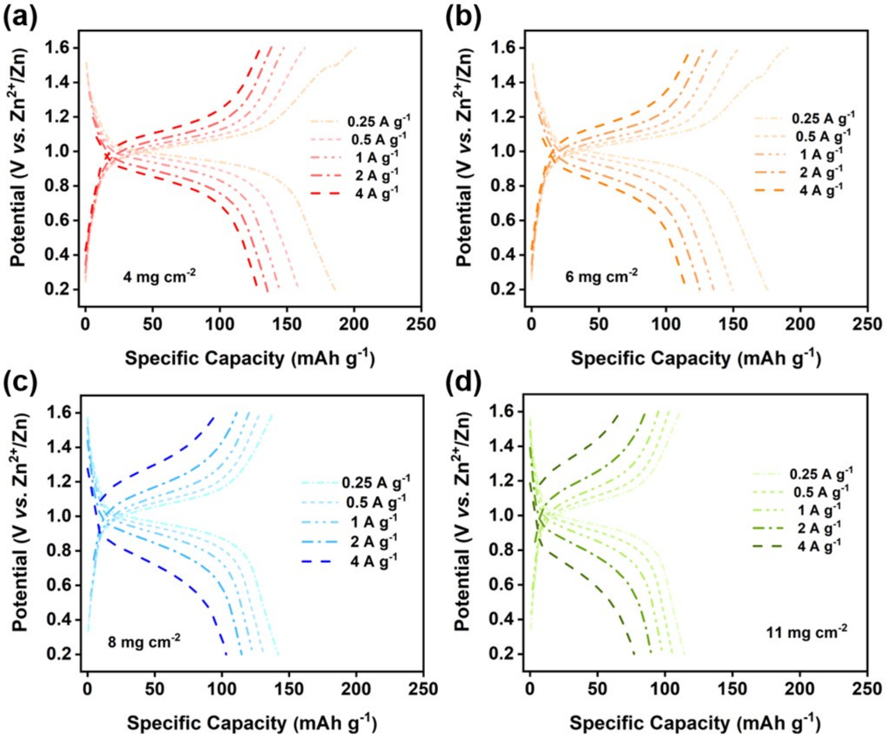

Fig. S14. GCD curves of C8Q cathodes with different active material loadings of (a) 4, (b) 6, (c) 8, and (d) 11 mg cm^{-2} in 3.0 M Zn(OTf)$_2$ at various current densities.

16. The cycle performance of the C8Q cathodes with different active material loadings in 3.0 M $Zn(OTf)_2$ at 4 A g^{-1}.

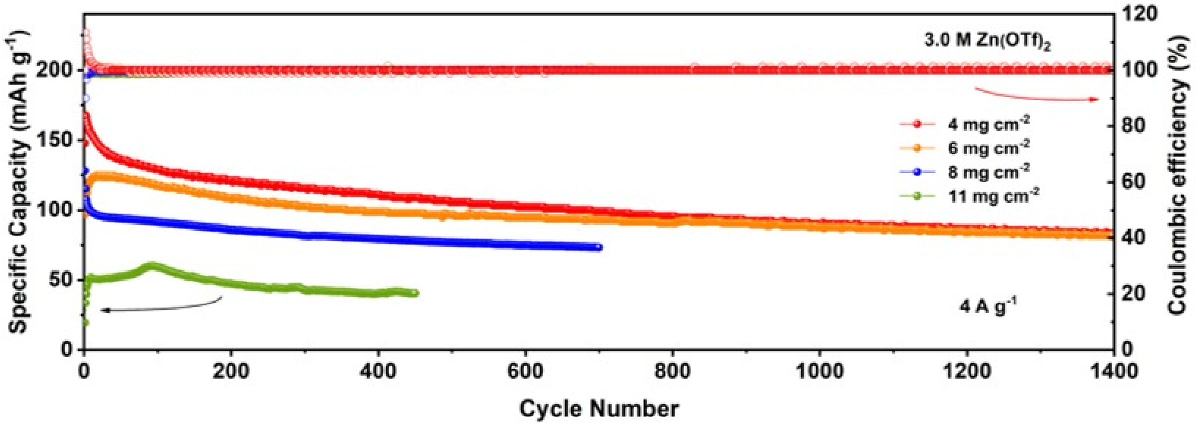

Fig. S15. Cycle performance of C8Q cathodes with different active material loadings of 4, 6, 8, and 11 mg cm^{-2} in 3.0 M $Zn(OTf)_2$ at 4 A g^{-1}.

17. The logarithmic relationship of the C8Q cathode between the peak currents and the scan rates.

The relationship between current (mA) and scan rate (mV s^{-1}) can be expressed as Equations (1) and (2):

$$i = av^b \quad (1)$$

$$log(i) = blog(a) + log(a) \quad (2)$$

18. The GITT test.

During the GITT test, the battery was discharged and charged with a pulse current of 50 mA g^{-1} for 20 minutes between rest intervals for 60 minutes. The experimental potential ranges were 0.2-1.6 V. The Zn^{2+} ion diffusion coefficient could be calculated by the following equation:

$$D = \frac{4}{\pi\tau}\left(\frac{n_m V_m}{S}\right)^2\left(\frac{\Delta E_s}{\Delta E_t}\right)^2 \quad (3)$$

where τ is the duration of the current pulse (s) of Zn^{2+}, S is the contact area between the cathode and electrolyte (cm^2), n_m is the amount of substance (mol), and V_m represents the molar volume of the C8Q molecule. ΔE_s and ΔE_t are the voltage changes caused by the pulse and charge-discharge processes, respectively.

19. The XPS spectra of C8Q cathodes at different charge and discharge states.

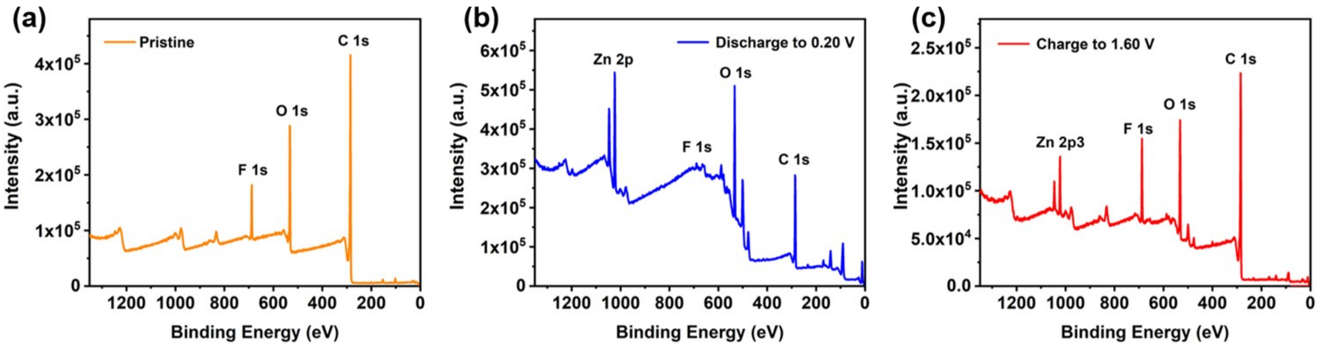

Fig. S16. XPS spectra of C8Q cathodes at different states: (a) pristine, (b) discharged to 0.20 V, and (c) recharged to 1.60 V.

20. The XRD characterization of C8Q cathodes at different states.

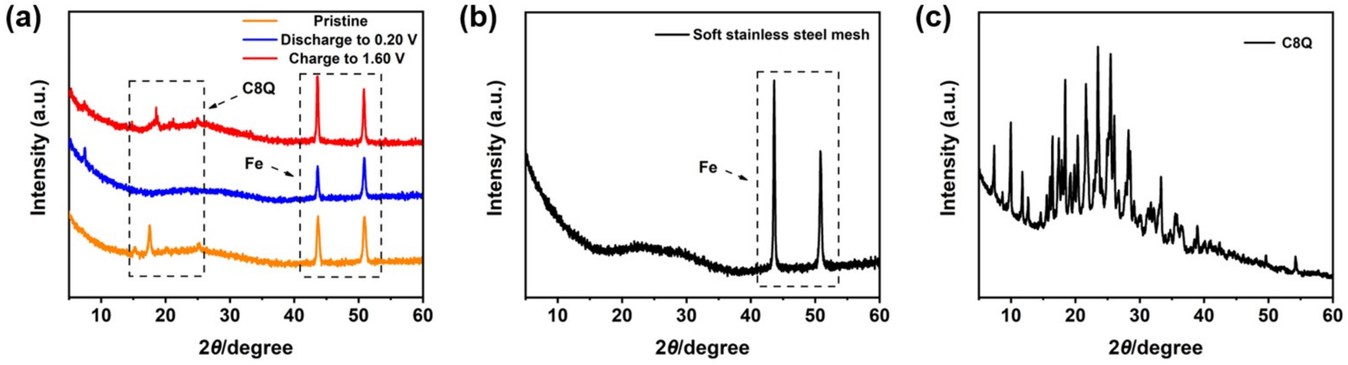

Fig. S17. Ex-situ XRD patterns of (a) C8Q cathodes at different states: pristine, discharged to 0.20 V, and recharged to 1.60 V. (b) the XRD pattern of soft stainless steel mesh. (c) the XRD pattern of C8Q.

21. The CV curves of the C8Q cathode in 3.0 M $Zn(OTf)_2/H_2O$ (pH=4.35), CF_3SO_3H/H_2O (pH=4.35), saturated $Zn(OTf)_2$/acetonitrile (AN) electrolyte measured by typical three-electrode systems.

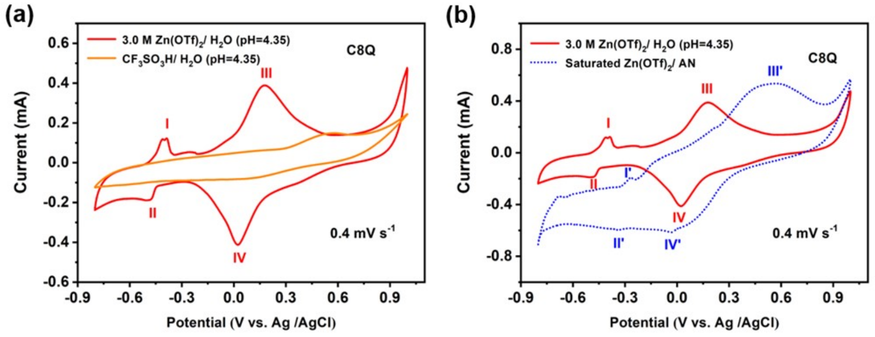

Fig. S18. CV curves of the C8Q cathode measured by typical three-electrode systems: (a) 3.0 M $Zn(OTf)_2/H_2O$ (pH=4.35) and CF_3SO_3H/H_2O (pH=4.35) electrolyte. (b) 3.0 M $Zn(OTf)_2/H_2O$ (pH=4.35) and saturated $Zn(OTf)_2$/AN electrolyte.

22. The assembly of the flexible belt-shaped Zn||C8Q battery.

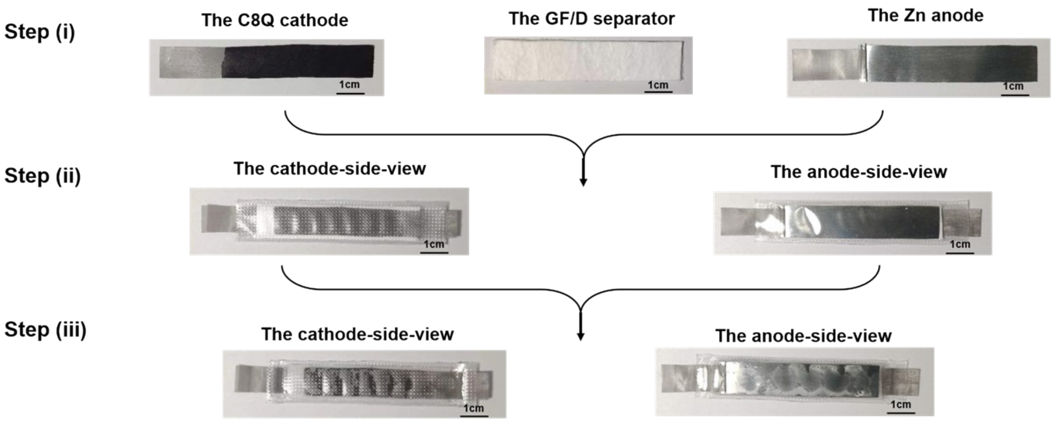

Fig. S19. Assembly process of the flexible belt-shaped Zn||C8Q battery: Step (i) Preparation of electrodes and diaphragm; Step (ii) Assembly; Step (iii) Perfusion of the electrolyte and seal of batteries.

23. The assembly of the flexible belt-shaped Zn||C8Q battery.

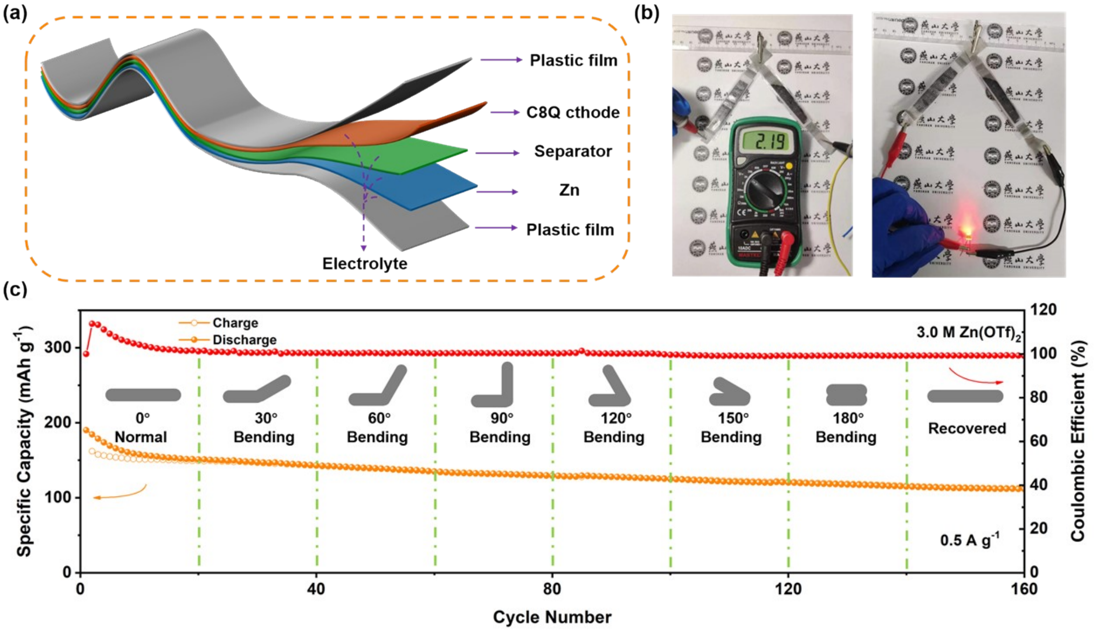

Fig. S20. (a) Structural diagram of the flexible belt-shaped Zn||C8Q battery. (b) Practical demonstrations of lightening an LED light using such batteries. (c) Cycle stability at successive various repeated bending states at a current density of 0.5 A g^{-1}.

References

1 C. Li, W. Yuan, C. Li, H. Wang, L. Wang, Y. Liu and N. Zhang, *Chem. Commun.*, 2021, **57**, 4319-4322.

2 B. W. Olbasa, C. J. Huang, F. W. Fenta, S. K. Jiang, S. A. Chala, H. C. Tao, Y. Nikodimos, C. C. Wang, H. S. Sheu, Y. W. Yang, T. L. Ma, S. H. Wu, W.N. Su, H. Dai and B. J. Hwang, *Adv. Funct. Mater.*, 2022, **32**, 2103959.
